# Varying impact of neonicotinoid insecticide and acute bee paralysis virus across castes and colonies of black garden ants, *Lasius niger* (Hymenoptera: Formicidae)

**DOI:** 10.1038/s41598-021-98406-w

**Published:** 2021-10-15

**Authors:** Daniel Schläppi, Nina Kettler, Gaétan Glauser, Lars Straub, Orlando Yañez, Peter Neumann

**Affiliations:** 1 Institute of Bee Health, Vetsuisse Faculty, University of Bern, Bern, Switzerland; 2 School of Biological Sciences, University of Bristol, Bristol, UK; 3 Neuchâtel Platform of Analytical Chemistry, University of Neuchâtel, Neuchâtel, Switzerland; 4 Swiss Bee Research Centre, Bern, Switzerland

**Keywords:** Behavioural ecology, Community ecology, Conservation biology, Ecosystem services, Ecology, Zoology, Entomology

## Abstract

Pesticides and pathogens are known drivers of declines in global entomofauna. However, interactions between pesticides and viruses, which could range from antagonistic, over additive to synergistic, are poorly understood in ants. Here, we show that in ants the impact of single and combined pesticide and virus stressors can vary across castes and at the colony level. A fully-crossed laboratory assay was used to evaluate interactions between a sublethal dose of the neonicotinoid thiamethoxam and Acute bee paralysis virus (ABPV) in black garden ants, *Lasius niger*. After monitoring colonies over 64 weeks, body mass, neonicotinoid residues and virus titres of workers and queens, as well as worker behavioural activity were measured. ABPV, but not thiamethoxam, reduced activity of workers. Neonicotinoid exposure resulted in reduced body mass of workers, but not of queens. Further, thiamethoxam facilitated ABPV infections in queens, but not in workers. Overall, virus exposure did not compromise detoxification and body mass, but one colony showed high virus titres and worker mortality. Although the data suggest additive effects at the level of individuals and castes, co-exposure with both stressors elicited antagonistic effects on colony size. Our results create demand for long-term holistic risk assessment of individual stressors and their interactions to protect biodiversity.

## Introduction

The global entomofauna is affected by precipitous declines in both abundance and diversity^1–3^. This is of major concern because insects play indispensable roles in terrestrial ecosystems. They provide essential regulating and supporting ecosystem services of high economic value and crucial for food security, such as pollination, natural pest control, maintenance of soil fertility and soil formation^4,5^. Consequently, the reported declines are alarming and jeopardize the functions and services provided by insects^6,7^. The main drivers are habitat loss and fragmentation, anthropogenic pollution, invasive species, pathogens and climate change^8–10^. Furthermore, there is growing consensus that no single stressor is driving current declines, but rather complex interactions amongst a plethora of intertwined stressors^8,11,12^.

Today’s human food production profoundly relies on agro-chemicals, which represent one major anthropogenic pollution factor that acts as a known driver of insect declines^8,13^. The prophylactic abundant usage of non-specific pesticides to combat pest species inevitably harms non-target organisms^14,15^. As systemic pesticides, neonicotinoids are usually applied as seed coatings and only a minor fraction of the active ingredients is actually taken up by the plant roots, resulting in widespread environmental contaminations^16,17^. Even though environmental concentrations of neonicotinoids are often low, they can persist for a long time in the environment as they often have long half-life times (DT_50_)^18^. For example, the DT_50_ of thiamethoxam ranges between 7 and 92 days in field studies or between 34 and 233 days in laboratory studies^19–21^. For clothianidin, a metabolite of thiamethoxam^22^, the degradation is even slower with the field DT_50_ in the range of 13.3–305.4 days and DT_90_ ranging from 188 to more than 1000 days^20,21^. Chronic exposure of soil-dwelling organisms to fluctuating concentrations and mixtures of neonicotinoids in the range of 1 to > 100 ppb in arable farmlands are likely^17,18^. Therefore, they may induce long-term inadvertent sublethal effects on insect physiology and behaviour^7,14,15^. Harmful impacts on cognitive abilities, foraging behaviour, immune functions, colony development, fertility and reproductive output have already been reported to influence insects at individual, colony and community levels^16,23,24^. Of particular concern is a reduction in fitness, as this ultimately governs the state of all wild populations^25^. Social insects, which are to be considered among the most important insects from an ecological perspective^26^, are unsurprisingly also affected by exposure to neonicotinoid insecticides^24,27,28^.

Colonies of eusocial insects are characterized by overlapping generations, cooperative brood care and reproductive division of labour between castes, whereby one or a few female individuals (queens) reproduce, and the rest of the usually non-reproductive females (workers) perform all other tasks necessary to maintain the colony^26^. These colonies are superorganisms, with individual insects being analogous to cells in multicellular organisms, acting as a functional unit^29,30^. Consequently, when studying the effects of stressors on eusocial insects, it is crucial to consider life-history traits specific to such superorganisms^26–30^. Therefore, a holistic understanding incorporates effects ranging from the individual, over the caste to the colony level, which in turn needs to be differentiated according to life-stages or caste-specific exposure risks and susceptibilities^28,31^. At an individual level, subtle effects of chronic exposure to sublethal insecticide doses can often be difficult to detect, yet over time they may translate into negative long-term consequences affecting a colony’s fitness^32^.

Ants are ubiquitous social insects crucial for ecosystem functioning as they provide essential ecosystem engineering and services such as natural pest control and pollination^33,34^. The combination of the long persistence of neonicotinoids^18–21^, perennial sedentary colonies and long life-spans, which characterize queens of many ant species, make long-term pesticide exposure highly likely^27,28,35^. Furthermore, long-term exposure inevitably leads to co-exposure and potential interactions with other stressors. Although interactions amongst stressors are often assumed to be synergistic per se, they may be additive or even antagonistic depending on the nature of the stressors and susceptibility of an organism^36,37^, which in turn underlies inter- and intra-species specific variability^31^. Factors such as age, developmental stage or sex influence stressor susceptibility leading to within-species differences^31,38^. While varying levels of susceptibility have been shown for haploid male (drone) and diploid female (worker) honey bees, data comparing queens and workers remain scarce^31^. In most species, queens and workers exhibit pronounced differences in morphology and longevity, and queens appear to be more tolerant of toxins^27,33,39^. Consequently, it seems plausible that susceptibility and stressor interactions vary between the two castes.

Interactions between pesticides and pathogens have recently received considerable attention^40,41^. Sublethal doses of pesticides may act as immune suppressors, thereby enhancing the spread and deleterious effects of pathogens^42–44^. For example, thiamethoxam and its metabolite clothianidin have been shown to affect the immunocompetence of honey bees, potentially enhancing susceptibility to viruses^45–47^. Viral agents affecting the health of Western honey bees (*Apis mellifera*) are well known^48^. However, with the rise of the invasive ectoparasitic mite *Varroa* *destructor*, the honey bee virus landscape has changed drastically^49,50^. This potent virus vector drives prevalence, titres and adaptions of viruses associated with it^49–51^. With elevated titres of these emerging infectious diseases in the apiary environment, the probability of host shifts increases^52^, ultimately affecting the wider insect community^53,54^. Viruses first described in honey bees, such as the two positive-sense single-stranded RNA viruses Deformed wing virus and Acute bee paralysis virus (ABPV), are now considered multi-host pathogens with a broad host range, including ants^53–58^. However, to our knowledge, no study has yet addressed the co-exposure to pesticides and viruses in ants^28,41^.

Here, we take advantage of recent findings that black garden ants, *Lasius niger*, are alternative hosts of ABPV, which can display clinical symptoms^58^. These common ants, endemic to Europe, can exploit all food resources known for ants except for direct granivory, and as ubiquitous generalist predators as well as being prey for other animals they play an important role in food webs^59^. Since they frequently scavenge on honey bees, exposure to honey bee viruses is likely and indeed there is a high prevalence of viruses in ants sampled in the surroundings of apiaries^58,60^. *L. niger* is characterized by medium to small-sized usually subterranean colonies in cities, gardens, meadows and arable lands^59^. Usually, the colonies are monogyne with extremely long-lived queens^59^ (up to 28 years^61^). Therefore, black garden ants represent an ideal model system to study potential interactions between long-term neonicotinoid insecticide exposure and viruses. We investigated sublethal effects of the neonicotinoid insecticide thiamethoxam and ABPV on *L. niger* at the colony and individual queen/worker level in a fully-crossed laboratory experiment. Thiamethoxam was chosen because it is amongst the most frequently used neonicotinoid insecticides^62^, and because it is metabolized into an active insecticide after entering the host, i.e. its primary metabolite clothianidin^22^. Over 64 weeks, newly founded colonies were chronically exposed to thiamethoxam (at a dose of 30 ppb^18,63,64^) or not (controls). Additionally, within each group, colonies were fed for 10 weeks with artificially ABPV infected honey bee pupae (*A. mellifera*) or control pupae. Workers were tested in a behavioural assay and we measured colony sizes, as well as neonicotinoid residue concentration, virus titres and body mass in both castes. Residue concentrations of both thiamethoxam and clothianidin were analysed to estimate levels of uptake and detoxification^27^.

In bees, clinical symptoms induced by ABPV include quickly progressing paralysis trembling movements, inability to fly, loss of hairs on thorax and abdomen, gradual darkening appearance and adult mortality^65^, and in ants a reduced movement speed and impaired locomotion have been described^58^. Further, ABPV infections can result in decreased colony size^58,65^. Sublethal effects of neonicotinoid insecticides on social insects include impaired locomotion, changes in activity, negative impacts on cognitive abilities, fertility, colony growth, reproductive output and effects on the immunocompetence^66^. Based on current literature, we therefore expected that both the neonicotinoid and the virus would affect the behaviour of workers and colony development and that thiamethoxam facilitates viral infections due to a compromised immune system, thereby resulting in a synergistic interaction of the two stressors.

## Results

### Survival, body mass and colony strength

Overall, queen mortality (i.e. colony failure) was 20% until the end of the experiment (week 64). There was no significant difference among the four treatments (controls, neonicotinoid = chronic exposure to thiamethoxam (30 ppb), virus = feeding regime with ABPV, combined = exposure to thiamethoxam and ABPV; each N = 10; log-rank test, *X*^2^ = 0.0, df = 3, *p* = 1.0). Overall, low worker mortality was observed, but it was not further traced in detail because dead ants were deposed on waste piles and therefore hardly recognisable^67^. However, at the end of the feeding regime (week 61), 37 workers were found lying dead in the foraging arena of one colony from the combined treatment. Six of the 40 queens were not able to raise workers successfully, i.e. not a single worker emerged, despite laying eggs (control and virus one queen each, neonicotinoid and combined treatment two queens each), but neither of the two treatment factors (neonicotinoid—*X*^2^_(1)_ = 0.78, *p* = 0.37, virus—*X*^2^_(1)_ = 0, *p* = 1.0) nor the interaction term (virus:neonicotinoid—*X*^2^_(1)_ = 0.0, *p* = 1.0) had a significant effect (N = 10 per treatment).

**Figure 1:**
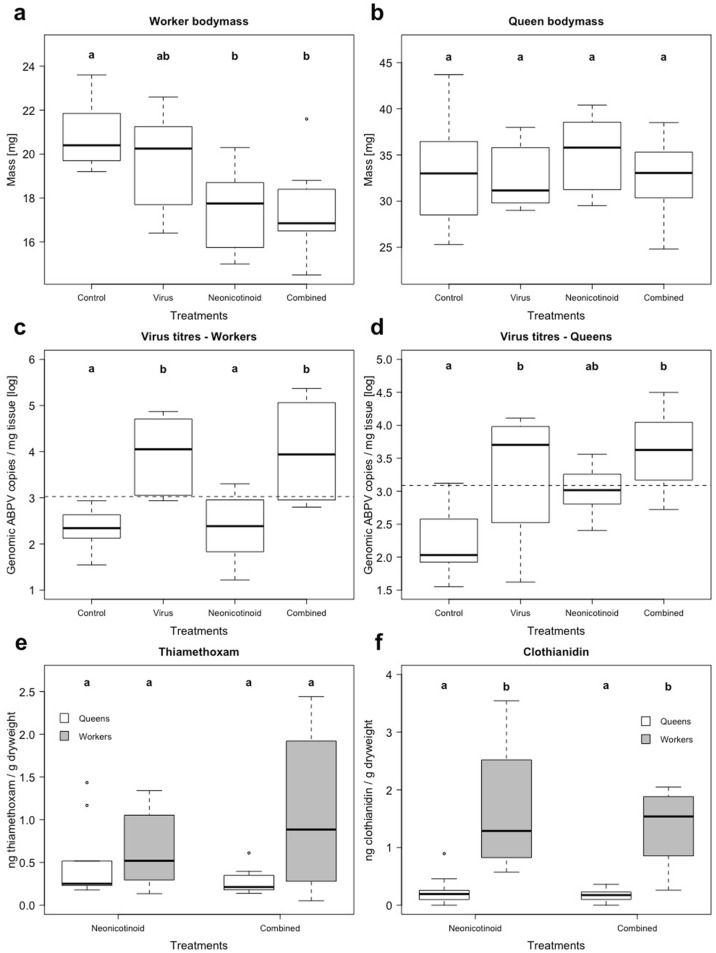
Body mass, virus titres and neonicotinoid residues of queens and workers, *Lasius niger.* Body mass (mg) of 20 pooled workers (**a**) and queens (**b**) as well as genomic copies of Acute bee paralysis virus (ABPV) [log] per mg tissue of workers (**c**) and queens (**d**) under the following four treatments groups: Controls, neonicotinoid = chronic exposure to thiamethoxam (30 ppb), virus = feeding regime with ABPV and combined = exposure to both thiamethoxam and ABPV. Neonicotinoid residues [ng/g dry mass] of queens (white boxplots) and workers (grey boxplots) in the neonicotinoid and combined treatment are depicted in panels (**e**) for thiamethoxam and (**f**) for clothianidin. Boxplots with inter-quartile-ranges, medians and outliers as well as the virus detection thresholds for both castes (dashed line) are shown as well as significant differences (p < 0.05) between treatments, which are indicated by bold letters (Tukey post hoc test).

The body mass of workers was significantly affected by neonicotinoid exposure (N = 8 per treatment, *F*_(1)_ = 16.7, *p* < 0.001), while neither the virus exposure nor the interaction term showed significant effects (virus—*F*_(1)_ = 0.74, *p* = 0.4; interaction—*F*_(1)_ = 0.71, *p* = 0.41; Fig. 1a; Table 1). The post hoc pairwise comparison revealed that workers from the neonicotinoid and the combined treatment had a significantly lower body mass compared to the controls (*p* < 0.01, Fig. 1a), while workers from the virus treatment were not significantly different from any other treatment group. The body mass of queens was not affected by either of the treatment factors or their interaction (N = 8 per treatment, neonicotinoid—*F*_(1)_ = 0.4, *p* = 0.53; virus—*F*_(1)_ = 0.91, *p* = 0.34; interaction—*F*_(1)_ = 0.38, *p* = 0.54; Fig. 1b).

**Table 1:** Summary table of response variables. Mean ± standard deviation values for colony strength (week 13 & 64) measured as number of workers and amount of brood (eggs, larva and pupae), mass of *Lasius niger* queens (♀) and 20 pooled workers (☿), virus titres [log genomic virus copies / mg] and neonicotinoid residues [ng/g_(dryweight)_] for the four treatment groups: (i) Controls, (ii) neonicotinoid = chronic exposure to thiamethoxam (30 ppb), (iii) virus = feeding regime with Acute bee paralysis virus (ABPV), (iv) combined = exposure to thiamethoxam and ABPV**.** N = 8 each unless specified differently in square brackets [].

Treatment		Control	Virus	Neonicotinoid	Combined
Colony strength week 13	# ☿	13.1 ± 6.72 [19]	-	14.1 ± 6.47 [18]	-
	# brood	37 ± 14.1 [19]	-	34.6 ± 13.8 [18]	-
Colony strength week 64	# ☿	286.8 ± 60.7	193.9 ± 61.3	161.3 ± 32.4	150.4 ± 52
	# brood	121 ± 57	59.5 ± 25.5	38 ± 18.2	36.1 ± 12.8
Mass	☿	20.8 ± 1.54	19.7 ± 2.2	17.5 ± 1.85	17.4 ± 2.09)
	♀	33.1 ± 5.98	32.6 ± 3.56	35.1 ± 4.24	32.6 ± 4.29
Virus titres	☿	2.34 ± 0.423	3.91 ± 0.88	2.36 ± 0.73	4.01 ± 1.10
	♀	2.22 ± 0.506	3.27 ± 0.93	3.02 ± 0.36	3.61 ± 0.61
Neonicotinoid residues					
Thiamethoxam [ng/g]	♀	0 ± 0	0.13 [1]	0.52 ± 0.47	0.27 ± 0.15
	☿	0 ± 0	0 ± 0	0.65 ± 0.45	1.08 ± 0.92
Clothianidin [ng/g]	♀	0 ± 0	0.14 [1]	0.25 ± 0.28	0.16 ± 0.11
	☿	0 ± 0	0 ± 0	1.67 ± 1.07	1.36 ± 0.67

**Figure 2:**
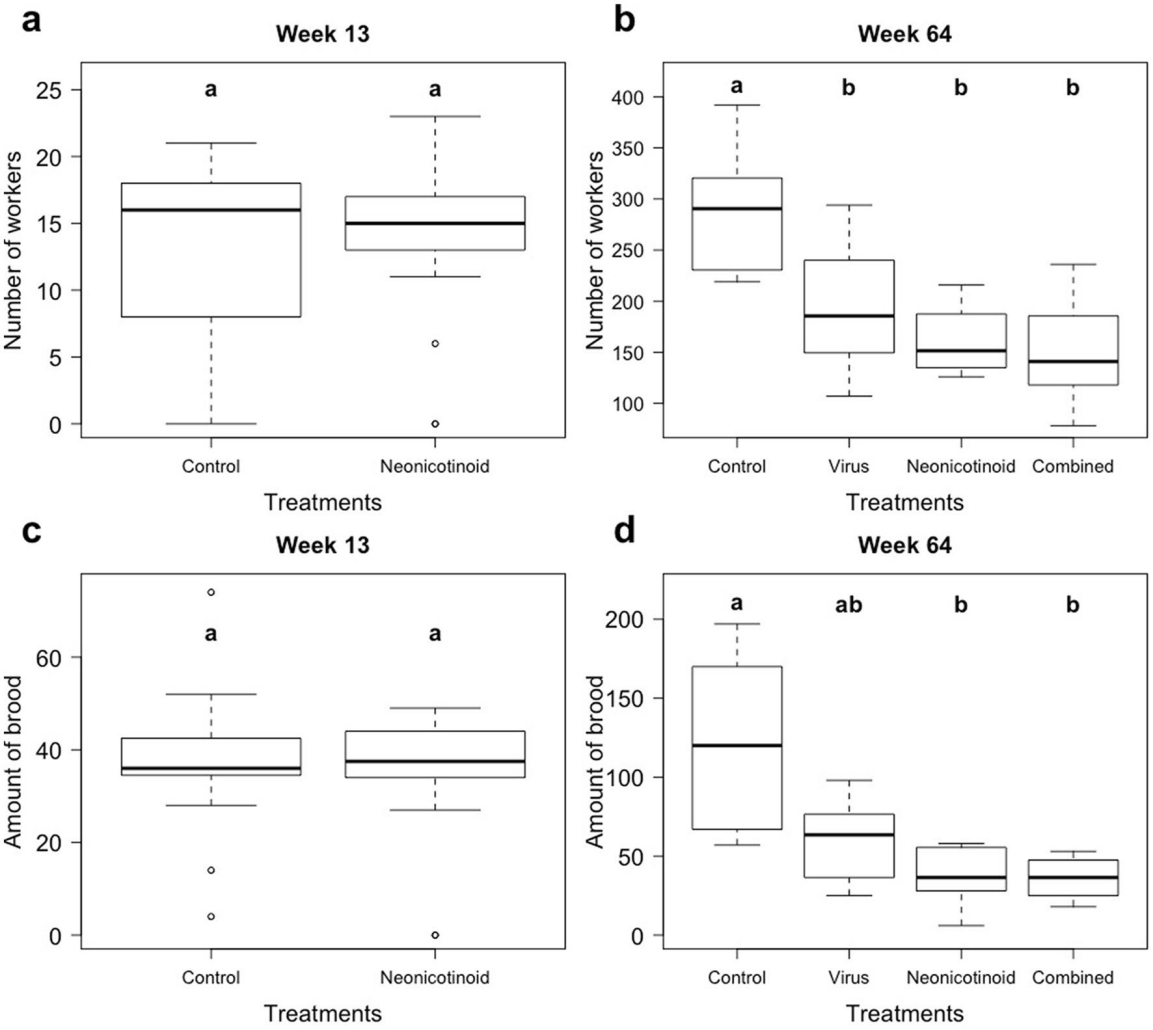
Colony strength. The number of *Lasius niger* workers (**a**, **b**) and the amount of brood (sum of eggs, larva and pupae; **c**, **d**) before the first overwintering (week 13, **a**, **c**) and the end of the experiment (week 64, **b**, **d**). At the first overwintering only the neonicotinoid treatment (chronic exposure to 30 ppb thiamethoxam) was established (controls N = 19, neonicotinoid N = 18). Colonies were then split in a stratified random way to two more treatment groups (each N = 8; virus = feeding regime with Acute bee paralysis virus (ABPV) & combined = exposure to thiamethoxam and ABPV. The inter-quartile-ranges, medians and outliers of boxplots are shown with significant differences (Kruskal Test (**a**, **c**), Tukey post hoc test (b&d); p < 0.05) between treatments being indicated by bold letters.

Before the first overwintering (week 13), there were no significant differences in colony strength measured as the number of adults (*X*^2^_(2)_ = 0.36, *p* = 0.83, Fig. 2a) and the amount of brood produced between the two treatments (control N = 19, neonicotinoid N = 18; *X*^2^_(2)_ = 0.004, *p* = 0.95, Fig. 2c). However, at the end of the experiment (week 64), both the number of workers and the amount of brood differed significantly among the four treatment groups (Fig. 2b,d): There were significant effects of the two treatments and an antagonistic interaction was revealed for both workers and brood (workers: neonicotinoid—*F*_(1)_ = 20.4, *p* < 0.001, virus—*F*_(1)_ = 7.7, *p* = 0.01, antagonistic interaction—*F*_(1)_ = 4.8, *p* = 0.037; brood: neonicotinoid—*F*_(1)_ = 21.9, *p* < 0.001, virus—*F*_(1)_ = 6.6, *p* = 0.015, antagonistic interaction—*F*_(1)_ = 7.0, *p* = 0.013; N = 8 per treatment). Post hoc pairwise comparison revealed that the controls had significantly more workers than the three treatment groups (all *p*’s < 0.01), which did not significantly differ among each other (all *p*-values > 0.37). Furthermore, controls had significantly more brood than the colonies from the neonicotinoid (*p* < 0.01) and the combined treatment (*p* < 0.01) , while the other treatment pairs were not significantly different amongst each other (all *p*-values > 0.29).

### Neonicotinoid residues

UHPLC-MS/MS analyses confirmed the absence of both thiamethoxam and clothianidin from all samples of the control and the virus treatment, except for one queen from the virus treatment (Table 1). In the other two treatment groups the residue concentration of thiamethoxam per gram dry mass was significantly affected by the factor caste (*F*_(1)_ = 7.3, *p* = 0.016) while neither the virus treatment nor the interaction term (caste:virus) showed significant effects (*F*_(1)_ = 0.43, *p* = 0.52; *F*_(1)_ = 3.48, *p* = 0.08; N = 8 per group; Fig. 1e). The post hoc pairwise comparison did not reveal significant differences among the four groups (all *p*-values > 0.23). A significant caste effect was determined for clothianidin residue concentrations (*F*_(1)_ = 89.5, *p* < 0.01), while virus and the interaction term had no significant effect (*F*_(1)_ = 0.25, *p* = 0.63; *F*_(1)_ = 0.09, *p* = 0.76; N = 8 per group; Fig. 1f). Queens had significantly lower concentrations of clothianidin compared to workers (all pairwise *p*-values < 0.05), but there was no difference between queens or workers of the different treatments. Further, the queens had a significantly lower ratio of clothianidin to thiamethoxam ratio compared to workers (*X*^2^_(1)_ = 17, *p* < 0.01).

### Virus analyses

Infection status (positive vs. negative) of workers were affected by virus exposure (*X*^2^_(1)_ = 32.9, *p* < 0.01), while thiamethoxam exposure and treatment interaction had no significant effect (neonicotinoid—*X*^2^_(1)_ = 3.06, *p* = 0.08; interaction—*X*^2^_(1)_ < 0.001, *p* < 0.99; N = 8 per treatment). No worker sample from the control treatment was tested positive for ABPV. In contrast, all samples of the virus and the combined treatment were positive, and interestingly two out of eight neonicotinoid treatment samples were positive (Table 1). Likewise, virus titres found in workers were significantly affected by virus exposure, yet neither neonicotinoid exposure nor the interaction term revealed significant effects (virus—*F*_(1)_ = 29.6, *p* < 0.01; neonicotinoid—*F*_(1)_ = 0.02, *p* = 0.9; interaction—*F*_(1)_ = 0.03, *p* = 0.86; Fig. 1c). The virus titre of workers that were found dead in one colony of the combined treatment (week 61) was 7.15 log virus copies per mg tissue (sixty times higher than any other ant sample).

In queens, six out of eight were positive in the virus treatment, four out of eight in the neonicotinoid treatment, seven out of eight in the combined treatment and all controls were negative (Table 1). The infection status of queens was significantly affected by virus and neonicotinoid exposure (virus—*X*^2^_(1)_ = 13.74, *p* = 0.95; neonicotinoid—*X*^2^_(1)_ = 4.66, *p* < 0.01) but no interactive effect was detected (*X*^2^_(1)_ = 2.52, *p* = 0.11; N = 8 per treatment). The same holds true for virus titres in queens (virus—*F*_(1)_ = 13.4, *p* < 0.01; neonicotinoid—*F*_(1)_ = 6.42, *p* = 0.02; interaction—*F*_(1)_ = 1.02, *p* = 0.86, additive effect; Fig. 1d). Queens and workers were not significantly different with regards to virus titres when standardized for body mass differences (*X*^2^_(1)_ = 0.42, *p* = 0.51), thus suggesting no significant caste effect.

### Behavioural assay

**Figure 3:**
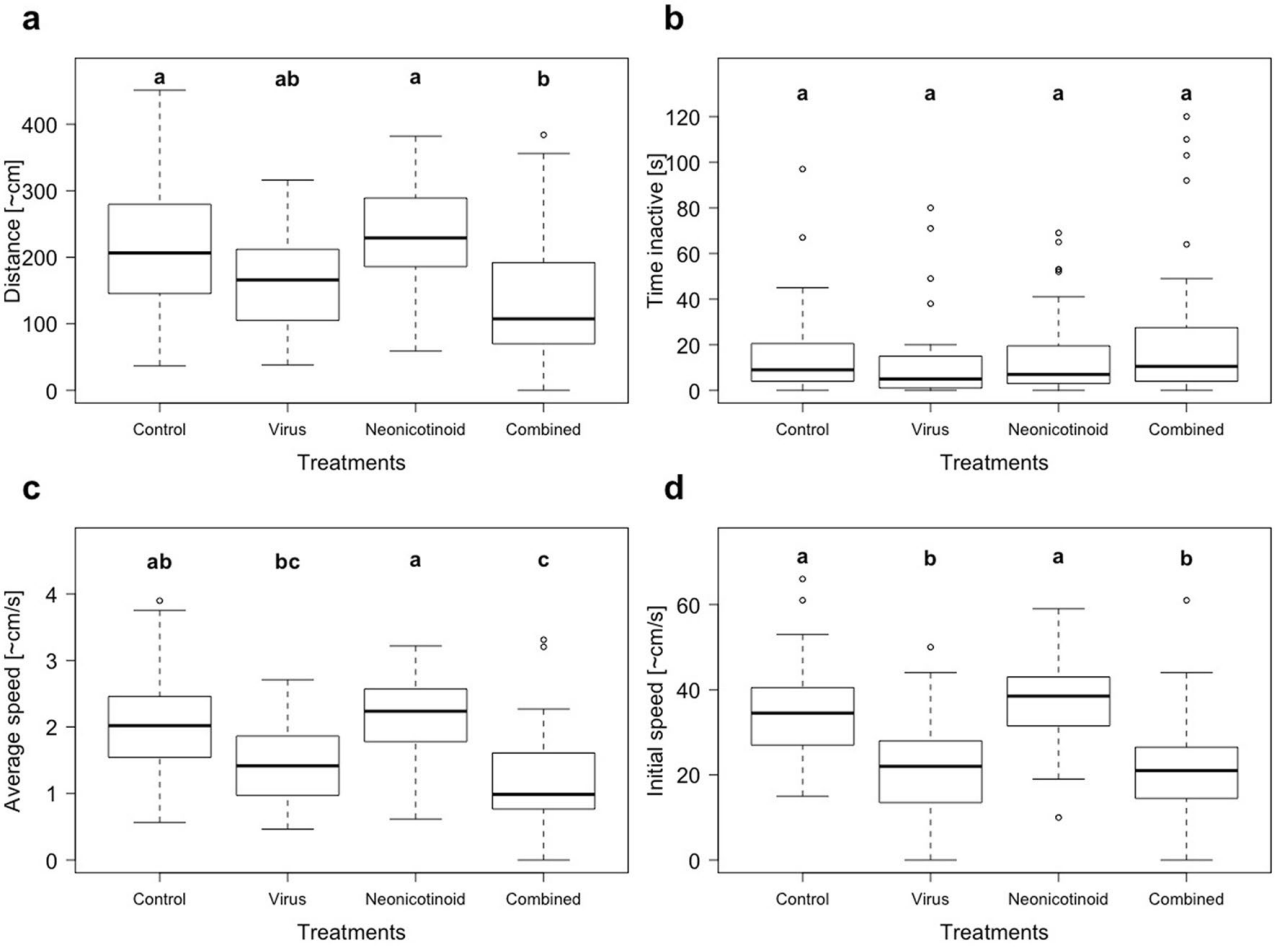
Behavioural assay. Movement analysis of *Lasius niger* workers from four different treatment groups (controls, neonicotinoid = chronic exposure to thiamethoxam (30 ppb), virus = feeding regime with Acute bee paralysis virus (ABPV), combined = exposure to thiamethoxam and ABPV) filmed for 120 s in a movement arena: (**a**) Estimated total distance covered, (**b**) amount of time the workers were inactive, (**c**) Average speed and (**d**) initial movement speed i.e. covered distance during the first ten seconds of observation. Significant differences between treatment groups is indicated by different letters (**a**, **b**; linear mixed effect modelling with post hoc Tukey correction, *p* < 0.05).

Virus exposure, but not neonicotinoid or the interaction, had a significant effect on overall movement (neonicotinoid—*X*^2^_(1)_ = 0.94, *p* < 0.33, virus—*X*^2^_(1)_ = 19.1, *p* < 0.01, interaction—*X*^2^_(1)_ = 1.99, *p* = 0.16; N = 40 per treatment). Pairwise comparison showed that workers form the controls and the neonicotinoid treatment covered significantly more distance compared to the workers of the combined treatment (both *p*’s < 0.01; Fig. 3a). No significant effects were observed regarding the amount of time workers were inactive during the observation time (neonicotinoid—*X*^2^_(1)_ = 1.24, *p* = 0.27, virus—*X*^2^_(1)_ = 0.57, *p* = 0.45, interaction—*X*^2^_(1)_ = 1.5, *p* = 0.22; N = 40 per treatment; Fig. 3b). Like overall movement, the average speed was significantly reduced by the factor virus but not the factor neonicotinoid or the interaction term (neonicotinoid—*X*^2^_(1)_ = 0.46, *p* = 0.5, virus—*X*^2^_(1)_ = 29.2, *p* < 0.01, interaction—*X*^2^_(1)_ = 2.26, *p* = 0.13; N = 40 per treatment). The controls were significantly faster than the workers of the combined treatment (*p* = 0.002) and the workers of the neonicotinoid treatment were faster than the workers of the virus and the combined treatment (*p* = 0.017, *p* < 0.01), but no more significant pairwise differences were revealed by the post hoc testing (*p*’s > 0.05; Fig. 3c). For the initial movement speed (i.e. in the first ten seconds) the effect of the virus exposure was even more pronounced (*X*^2^_(1)_ = 54.6, *p* < 0.01), while again, the factor neonicotinoid and the interaction term had no significant effects (*X*^2^_(1)_ = 0.04, *p* = 0.84; *X*^2^_(1)_ = 1.56, *p* = 0.21; N = 40 per treatment). Post hoc pairwise analysis revealed that workers of the control and the neonicotinoid treatment were significantly faster than the ones of the virus and the combined treatment (*p*’s < 0.05; Fig. 3d). Additionally, some workers of the virus and the combined treatment were observed to move unnaturally slowly, followed by uncontrolled trembling or twitching motions, or with partly immobile hind extremities, as described in Schläppi *et al*^54^. Neonicotinoid exposure had no effect on any of the measured behavioural variables and is therefore considered neutral in this context, and it also follows that no interactive effects were detected.

## Discussion

Our results show that co-exposure to a sublethal dosage of thiamethoxam and to ABPV can impact ants in a complex manner. Effects of the two stressors and their interaction vary, depending on the response variable and/or the tested level (i.e. individuals, castes, colonies). Although queen mortality and consequent colony failure are not affected, there is a clear effect at the colony level, where ABPV and thiamethoxam antagonistically affect colony strength. This implies that the colonies of the combined treatment do equally poor as the colonies exposed to individual stressors. Exposure to thiamethoxam appears to facilitate ABPV infections in queens, but not in workers. However, the exposure to virus seems not to affect the detoxification of the insecticide^27^. ABPV exposure affected locomotion and activity of workers, while no such effect was observed for thiamethoxam. This study highlights that the understanding of how ants and other social insects are impacted by multiple stressors requires consideration of acute and long-term effects, stressor interactions and effects at individual, caste and colony level.

Across all treatments, we observed low mortality of queens (i.e. colony failure) and workers. We can confirm that thiamethoxam at 30 ppb appears to have only sublethal effects on ants^27^, which matches the findings for imidacloprid^68^. Also, the queen’s ability to raise the first offspring was not affected. Even in the combined treatment, where one could expect increased stress, as the colonies are co-exposed to both stressors, no effects on either variable were detectable. Furthermore, we confirm that exposure to thiamethoxam reduces the body mass of workers, but not queens^27^. Body mass can be used as an indicator of health with potential implications on reproductive success^69,70^. Therefore, a reduction in body mass can be a symptom of an organism’s susceptibility towards a stressor^71^. In conclusion, the observed differences between queens and workers indicate that the latter are more susceptible towards thiamethoxam. In contrast, ABPV seems to have no effect on body mass irrespective of treatment or caste and no interactive effect with thiamethoxam was detected. However, exposure to ABPV resulted in impaired locomotion and reduced activity, confirming earlier results^58^: The distance covered, the average speed and the speed in the first seconds of observations were negatively affected by virus exposure. However, in contrast to our expectations, the behaviour of individuals was not affected by exposure to thiamethoxam. Moreover, no interactive effects of combined exposure were observed. In comparison, effects of neonicotinoid insecticide exposure on honey bee activity and locomotion have been shown, including longer inactivity, lower movement, and hyperactivity followed by hypoactivity^66,72^. Many aspects like foraging and competition (intra- and interspecific) are restricted by impaired locomotor abilities. For example, a reduced movement speed increases the time required to exploit available resources, which in turn can affect the colony growth rate^73^. A reduced work force impairs colony functioning, reduces competitive strength and decreases the number of produced sexuals^74–77^. Therefore, it is possible that the effects of virus exposure on locomotion can result in a reduction of colony performance and ultimately fitness (i.e. number of produced males and gynes that successfully contribute to the foundation of new colonies).

To determine possible caste specific differences in susceptibility towards ABPV, we used virus titres as an indication of infection levels. When normalized to body mass, there were no differences regarding the virus load of queens and workers. Despite the detection of clinical symptoms (i.e. impaired locomotion) the observed virus titres did not reach the levels of overt infections in honey bees^78^. However, there was one exception, as the workers found dead in one colony towards the end of the experiment reached more than 10^7^ copies per mg tissue. Why titres were higher in these specimens and why worker mortality was induced in this particular colony, but not in others, remains speculative. Our results suggest that ABPV infections can be lethal in *L. niger*, in addition to symptoms reported earlier^58^. A comparison of honey bees and ants is meaningful in this regard because these viruses have been studied in depth in the former^48^, but not in the latter. Both ants and honey bees are highly eusocial with reproductive division of labour, overlapping generations and cooperative brood care and therefore, they share key aspects of their life-history, which are clearly more important than other biological traits such as body size^26,33^. Indeed, comparisons of honey bees with solitary and even primitively eusocial bees (e.g. bumblebees) are not as appropriate despite obvious similarities in the pollinator guild^79^, due to superorganism resilience and other key features of eusociality^29,30^.

In queens, the data indicate that chronic exposure to thiamethoxam can result in higher virus titres. This suggests that the immune system may be weakened and the susceptibility towards ABPV increased, thereby supporting the idea that insect diseases can be linked to pesticides^43^. Clothianidin, the metabolite of thiamethoxam, modulates immune signalling, thus reduces immune defences and promotes virus replication in honey bees bearing covert infections^45^. However, the effect of thiamethoxam on the susceptibility towards ABPV were observed exclusively in queens, but not in workers. As the life expectancy of queens is much higher compared to workers, the former might invest more into detoxification, which in turn could negatively affect the immune response towards viruses. Another possible explanation could be that neonicotinoid insecticides interfere with the social structure of colonies or their ability to detect a pathogen, restricting social immune defences and thus increasing the chances of ABPV to reach the queen^80^. If or how neonicotinoids may affect the interaction network and social immunity in ants, as it has been suggested for bumblebees^32^, remains to be investigated. Some samples of the control and the neonicotinoid treatment being tested positive for the virus can perhaps be attributed to feeding with untreated honey bee pupae, which may have been infected by this virus. Indeed, although magnitudes lower than in artificially infected pupae, low background levels of viruses (i.e. covert infections) commonly occur in field-collected honey bee pupae^65^.

Further differences between the castes were revealed when looking at neonicotinoid residues to confirm uptake and metabolization of thiamethoxam. The residue levels indicate that queens seem to be able to cope better with the intoxication, indicating superior detoxification (as described in detail in Schläppi et al.^27^). This could also explain that thiamethoxam affects the body mass of workers but not of queens. Just as neonicotinoids facilitate viral infections^45^, we might expect an inverted effect where the induced stress of an ongoing virus infection vice versa affects detoxification negatively. However, exposure to ABPV had no detectable effect on the residue concentration of either thiamethoxam or clothianidin. This would be in line with findings in honey bees, where co-exposure to thiamethoxam and chronic bee paralysis virus resulted in an increase in virus loads, but neither the survival nor the ability to metabolize the insecticide to clothianidin was affected^46^. Noteworthy is the observation that one queen of a treatment group without thiamethoxam was tested positive for the insecticide. Given that no negative control was positive, thereby excluding cross-contaminations, the most parsimonious explanation is exposure in the field prior to sampling.

Our data revealed that the effect of thiamethoxam exposure on colony strength only becomes visible in the second year of colony development and that in colonies exposed to virus fewer adults emerged^27,58^. In contrast to our expectations, the colony strength (number of workers and brood) of the combined treatment was not different from the neonicotinoid and virus treatment. Thus, at colony level, the two stressors interact antagonistically, meaning that co-exposed colonies are doing better than expected if the individual effects of viruses and neonicotinoids are summed up^36,37,81^. These findings are in line with studies in bees suggesting that interactions between pesticides and pathogens are often antagonistic, particularly interactive effects on proxies of fitness such as behaviour, parasite load and immune responses^36,37^. Overall, these results highlight that the nature of stressor impacts and interactions strongly depends on the variable and effect level looked at. Examples such as the increase in susceptibility of queens towards ABPV when exposed to thiamethoxam demonstrate that effects at an individual level do not necessarily translate to colony level effects. When studying stressor impacts on social insects, it is thus crucial to consider colony-level effects. Superorganism resilience and social immunity are just two factors which show that effects on individuals have limited explanatory power when it comes to colony fitness^29,30^. A limitation of this study is that colony fitness was not assessed directly, as *L. niger* typically does not produce sexuals within the first years of colony development^33^. Therefore, we had to rely on colony size as a token of fitness in *L. niger*. Nonetheless, the observed effects are likely to affect colony fitness, because the number of workers and impaired locomotion affect many factors of colony functionality^74,75^, especially foraging as well as intra- and interspecies competition^76^. Lastly, the onset of the reproductive stage is earlier and more sexuals are produced in colonies with a larger workforce^77^. In this experiment, only a single concentration of thiamethoxam was tested, which is not sufficient for the demonstration of a dose–response or more complex response patterns such as dose-dependent synergism/antagonism^82,83^. Therefore, our results are only valid at the used neonicotinoid concentration and further experiments with a range of varying thiamethoxam concentrations would be required to draw definite conclusions about combined effects with ABPV.

The use of chronic exposure in the laboratory constitutes a conservative approach as there are no foraging risks due to predation, no competition and additional pathogens or unfavourable weather conditions. Furthermore, field colonies are usually exposed to multiple pesticides simultaneously, and exposure peaks that can occur during times of insecticide applications are absent in such a laboratory setting^84^. Hence, future studies should address how frequently and how long ants are exposed to stressors under field conditions and measure how high residues are in such specimen. Furthermore, the relative importance of multiple simultaneous exposure routes (acute and chronic) such as contact with spray droplets, contaminated soils, foliage, and water, as well as consumption of contaminated nectar, pollen, guttation fluid and seeds, honeydew, invertebrate prey and water are all poorly understood^28^. The exposure scenario in this laboratory study only covers exposure via contact with contaminated cotton reflecting contaminated soil and consumption of contaminated water reflecting soil pore water, neglecting additional exposure pathways. Uptake via consumption of water seems to be more important than topical contact with the toxicant because of the weak hydrophobicity of the neonicotinoids yielding a low penetration through the cuticula^66^. Another topic not addressed within the scope of this experiment is the question of bioavailability, i.e. what fractions of neonicotinoids present in soils are available for uptake, and for causing adverse effects to biota^85^. Therefore, the extent of the actual exposure of ants in contaminated soils remains elusive. Nonetheless, the neonicotinoid levels used in this experiment can be considered as a worst-case scenario as they were constantly in the upper range of what can be found in arable fields^17,18^. Furthermore, it can be assumed that such arable fields are probably less suitable habitats for ants in the first place because frequent disturbance, e.g. ploughing, will interfere with the construction of nest structures for perennial sedentary colonies. Nevertheless, ants frequently settle in arable lands^86^, and even if newly mated queens are successful in establishing a colony at the edge of such fields, long-term chronic exposure at lower dosages is likely^17^.

In conclusion, our results show that a holistic view is required to understand how an organism is affected by interacting stressors. Life-history traits, all levels of action, acute and long-term effects need to be considered as well as co-exposure to multiple stressors. In our case, it would not have been possible to estimate colony-level effects based on individual variables or if measurements were taken too early. Furthermore, effects could vary over time and therefore it would be necessary to monitor ant colonies over their entire life-cycle to understand the ultimate impact of any stressor. However, in current pesticide risk assessment schemes none of the indicator species for soil-dwelling insects are comparable to ants and other social insects^87–89^. Moreover, long-term effects as well as combined exposure scenarios with multiple pesticides and/or other stressors are mostly neglected so far^84,87–90^. Therefore, we emphasize the need to fully incorporate long-term effects, combined exposure scenarios and ants as model organisms in holistic future risk assessments. We argue that incorporating fitness as a key endpoint variable in future pesticide risk assessment schemes may substantially help to ensure more reliable and robust stressor evaluations^25^. This would subsequently mean that for eusocial insects the lifetime reproductive output of sexuals should be assessed either directly or with a reliable proxy in multi-generation laboratory essays to mitigate the future impacts of chemicals and other stressors on biodiversity^25^.

## Methods

### Experimental set-up

A fully-crossed laboratory experiment was established at the Institute of Bee Health, University of Bern, using *L. niger* colonies allocated to four treatment groups. The four treatment groups were (i) controls, (ii) virus—colonies fed with honey bee pupae artificially spiked with ABPV, (iii) neonicotinoid—colonies chronically exposed to thiamethoxam (30 ppb), and (iv) combined—colonies co-exposed to thiamethoxam and ABPV. No ethical approval was required to work with these invertebrate species and all experiments were performed in accordance with relevant guidelines and regulations. Gynes were collected in the field after their nuptial flights (30.07.2016 in Bern, Switzerland; for a detailed timeline see supplementary Fig. 1 and supplementary Table 1). Upon initiation of the experiment, 40 egg-laying queens were randomly allocated to two groups (Controls, Neonicotinoid; each N = 20). The second treatment started in July 2017 (week 51), where the groups were split in a stratified random way accounting for colony size, resulting in four treatment groups. Until the first overwintering (week 13), the colonies were kept in prepared nesting tubes (155 mm length, 14 mm inner diameter) wrapped in aluminium foil, maintained at RT (19–23 °C) and protected from direct sunlight^27^. After overwintering (week 34), the colonies were transferred into new nesting tubes with an attached foraging arena (135 × 68x32 mm, supplementary Fig. 2), where they were kept until the experiment was ended with freezing and storing of the colonies at − 80 °C prior to the second overwintering.

### Neonicotinoid exposure

The nesting tubes were separated by a sterile cotton wool ball into two compartments. The front compartment, closed by another cotton wool plug, housed the colony while the hind chamber was filled with 10 ml treatment solution^27^. Solutions were freshly prepared at the beginning of the experiment and after overwintering with Thiamethoxam (> 99.9% purity, Sigma-Aldrich, St. Louis, Missouri, USA) directly dissolved in distilled water. To test for the effects of chronic thiamethoxam exposure over the full experimental duration (64 weeks), a concentration of thiamethoxam was chosen (30 ppb) as it may be detected in soils of arable farmlands^18,63,64^. This concentration is below the level found in fields where the insecticides have just been applied but it is in the range of what can be found in fields up to 1 year post treatment^18^. Ultra-high performance liquid chromatography-tandem mass spectrometry (UHPLC-MS/MS) was used to confirm neonicotinoid persistence in pooled treatment solutions collected from the nesting tubes at the moment of nest translocation (week 34) and at the end of the experiment (week 64)^27^.

### Feeding regime

Before the first overwintering, colonies were provided twice with 30 μl droplets of sugar-water (50% mass fraction of sugar) once the first workers emerged, due to claustral colony founding^59^. Post hibernation, colonies received a sugar-water (40% mass fraction of sugar) drenched cotton ball and four fruit flies (*Drosophila hidey*) weekly. With the start of the treatment feeding regime, we switched from *Drosophila* to Western honey bee (*Apis mellifera*) pupae. The colonies in the virus treatments received honey bee pupae artificially spiked with ABPV, while the controls received control pupae for 10 weeks. For this, white-eyed honey bee pupae were obtained from sealed worker brood frames of local colonies (Bern-Liebefeld, Switzerland). Using standard methods^91^, half of the pupae were microinjected laterally between the second and third segment of the abdomen with 2 μL of an ABPV solution for virus propagation. Then, all pupae were incubated in darkness at 34.5 °C, ≥ 50% RH for 5 days. Virus presence in injected pupae was confirmed using quantitative real-time polymerase chain reactions (RT-qPCR; ABPV titres > 10^11^ genomic copies per pupae) and pupae were frozen at − 80 °C until needed.

### Parameter assessment

#### Colony size and body mass

The amount of brood (i.e. eggs, larvae and pupae) and the number of workers were assessed before the first overwintering (week 13) and at the end of the experiment after freezing the colonies (week 64). Further, the numbers of workers and pupae were counted at the start of the second treatment (week 51). The body mass of individual queens and twenty pooled workers was measured using an analytical balance (METTLER TOLEDO AT400; ± 0.1 mg) at the end of the experiment.

#### Behavioural assay

For each colony, the behaviour of five randomly chosen foragers sampled from the foraging arenas was analysed, as described in detail in Schläppi et al.^58^, 2 weeks after the feeding regime ended (week 63). In brief, the movement of individual ants in an arena (Θ = 90 mm) was video recorded for 120 s. Motion data were visually evaluated from slow motions recordings by quantifying the movement of ants in relation to a reference grid (grid cell: 8.5 mm × 8.5 mm), i.e. the number of grid cells passed over time. Only one grid cell was counted if an ant touched two cells at the same time, e.g. when walking along a line. Thereby, the overall movement, time inactive, average speed and initial speed was assessed. Overall movement was defined as the number of grid cells passed during the entire 120 s, time inactive as the time the ants did not move (including grooming and standing still), average speed as the number of cells passed in the time the ants were actively moving and initial speed as the number of cells crossed during the first 10 s. For more information and an exemplary video see Schläppi et al.^58^.

#### Neonicotinoid analyses

Queens and workers of all colonies that lasted until the end of the experiment (week 64; N = 8 per treatment) were analysed for both thiamethoxam and its metabolite clothianidin using UHPLC-MS/MS analysis as depicted in Schläppi et al.^27^. In brief, an adapted QuEChERS protocol was used to prepare and purify pooled samples with worker abdomens and individual abdomens of queens. Subsequently, an Acquity UPLC system (Waters, Milford, MA) coupled to a TQ-S triple quadrupole (Waters) were used for the analyses of the purified samples. Solutions in MeOH 25% at 0.005, 0.05, 0.5, 5 and 15 ng/ml, each containing internal standards (concentration of 5 ng/mL) were used for internal calibration to quantify thiamethoxam and clothianidin followed by linear regressions weighted by 1/x. We then normalised the samples to their mass and expressed them as ng/g dry mass for comparisons. The quantification detection limits were 20 pg/g of tissue for thiamethoxam and 40 pg/g for clothianidin. We included blank samples as negative controls.

#### Virus analyses

First, RNA was extracted from the queens and a pooled sample of 40 workers from each colony using standard methods^92^. However, only the head, thorax and extremities were used, as the abdomens were used for the neonicotinoid analyses. Each sample was manually crushed with a mortar in TN buffer (100 mM Tris, 100 mM NaCl, pH 7.6). Fifty microlitres of the homogenate were then used for the RNA extraction with a NucleoSpin RNA II kit (MACHEREY–NAGEL, Oensingen, Switzerland). 0.3 ng Ambion RNA Control 250 was added to each sample at the first extraction step to monitor the efficiency of the RNA purification and cDNA synthesis^93^. After elution in 30 µL of elution buffer, RNA was stored at − 80 °C. Then, a M-MLV RT Kit (Promega, Dübendorf, Switzerland) was used for reverse transcription by incubating the template RNA (0.05–0.25 μg) in a Thermocycler (Biometra, Analytik Jena, Jena, Germany) with random hexamer oligonucleotide (0.75 μL, 100 μM) and RNase-free water in a final volume of 17.75 μL for 5 min at 70 °C. For, the cDNA synthesis, 5 × Buffer (5 μL), nucleoside triphosphate (dNTP; 1.125 μL, 10 mM) and reverse transcriptase (M-MLV; 1 μL) were added to a final reaction volume of 25 μL and incubated it at 37 °C for 60 min. The obtained cDNA was diluted 1/5 and used for RT-qPCR to estimate ABPV titres using a KAPA SYBR FAST Universal qPCR kit (Kapa Biosystems, Wilmington, North Carolina, United States). A duplicate of each sample was run for both the targeted virus and the exogenous internal reference in a 12 μL reaction mix containing SYBR green reaction mix (6 μL), Milli-Q water (2.52 μL), diluted cDNA (3 μL) and each 0.24 μL of the forward and reverse primers (ABPV F6548—TCATACCTGCCGATCAAG, ABPV B6707—CTGAATAATACTGTGCGTATC, 197 bp^94^; RNA 250-F—TGGTGCCTGGGCGGTAAAG, RNA 250-B—TGCGGGGACTCACTGGCTG, 227 bp^93^). In addition, four ten-folds serial dilutions (10^−2^ to 10^−5^ ng) of purified PCR products used as standard curves and two no-template negatives were included on each plate. The reaction was processed in an ECO Real-Time PCR machine (Illumina, San Diego, California, United States) according to the following cycling profile: 95 °C for 3 min (incubation) followed by 40 cycles of 95 °C for 3 s (denaturation) and 57 °C for 30 s (annealing, extension and data collection). Fluorescence reading at 0.5 °C intervals between 55 °C and 95 °C was used to perform a melting curve analysis after the amplification for the verification of the specificity of the PCR products. RT-qPCR output data, standard curves and the experimental dilution factors were used to calculate an estimate of viral copies per sample (virus titres). To account the exponential distribution virus titres and for body mass differences between the castes, virus titres were log-transformed, normalized to the mass of the samples and expressed as “Log_10_ genomic copies / g dry mass” throughout the manuscript and for the statistical analyses. A sample was considered negative with zero viral copies if it had a shifted peak or no peak at all in the melting curve analysis. For both castes, Cq values of the negative samples were transformed to hypothetical virus titres and of the maximum value of these “negative titres” a titre detection threshold was obtained normalized to the castes mean bodyweight.

### Statistical analyses

R version 3.6.3 was used to run all statistical analyses and create Figs. ^95^. The Shapiro–Wilk test and the Levene’s test were used to test data and model residues for normal distribution and homogeneity of variances for the selection of statistical tests. Colonies were used as individual replicates unless specified differently and upon queen death, the respective colony was no longer included in further analyses. Consequently, sample sizes decrease from N = 10 per treatment upon initiation of the experiment to N = 8 per treatment at the end.

Queen survival was analysed with a log-rank test using the *survdiff* function from the R package survival^96^. The ability of queens to raise workers (binary response variable), i.e. if queens were able to lay eggs and attend eggs/larvae/pupae adequately so that at least one viable worker emerges (success or failure), was analysed using a generalized linear model with binomial distribution^97^. Both treatments (virus, neonicotinoid) and their interaction term (cross-product term—virus:neonicotinoid) entered the model as fixed effects. Queen and worker body mass were analysed using linear models with body mass of either caste entering the model as a response variable again with both treatments and their interaction as fixed effects. ANOVA was used as a likelihood ratio test to obtain *p-*values by comparing the model with the effect in question against the model without it. If a model revealed significant main effects, it was followed up by pairwise comparisons using Tukey’s Honest Significant Difference test, thereby controlling for multiple comparisons. Based on the definitions of an additive effects model, synergism or antagonism occur, when combined effect of multiple stressors is greater (synergism) or less than (antagonism) the sum of effects elicited by the individual stressors^81,82^. Therefore, if the interaction term is non-significant additive effects were assumed and else, if the interaction (cross-product term) of the two stressors was significant, a deviation from additive effects was assumed. In these cases, we consulted the model estimates to claim synergism (estimate of the cross-product term < 0; greater than additive effect) or antagonism (estimate of the cross-product term > 0; lesser than additive effect)^82^.

Colony strength, measured as the number of workers and the amount of brood (i.e. sum of eggs, larva and pupae) was analysed before the first overwintering and at the end of the experiment. Because of the nature of the data and as only the thiamethoxam treatment was established before the first overwintering, colony strength was compared between two groups (controls, neonicotinoid) using a Kruskal–Wallis test (non-parametric). The number of workers at the end of the experiment entered a linear model as response variable and was analysed with both treatments and their interaction as fixed effects. As above, post hoc pairwise comparison was performed using Tukey’s Honest Significant Difference test. The amount of brood (response variable) was analysed using an aligned rank transformation for nonparametric factorial analyses with same fixed effects using the *art* function from the R package ARTool^98^. For cross-factor pairwise comparisons, we followed the developer’s recommendations and used Mann–Whitney U tests on the original data for each treatment pair and then corrected the results for multiple comparisons using Holm's sequential Bonferroni procedure correction.

Infection status of queens and workers each were analysed using a generalized linear model with binomial distribution and virus titres were analysed with linear models as described above. To meet the model assumptions for workers the response variable virus titre was log-transformed. Furthermore, the relationship between virus titre (response variable) and ant caste (fixed effect) was analysed with treatments and colony identity as random factors using a linear mixed effect model with the *lmer* function of the R package lme4^99^. Colony identity was used as a random factor because samples of the same colony are not completely independent^97^.

To compare neonicotinoid residues (thiamethoxam or clothianidin) detected in queens and workers among the treatments we again used aligned rank transformation for nonparametric factorial analyses^92^. All samples of the control and virus treatment were negative except for one queen sample, which was positive for thiamethoxam and clothianidin. Non-positive negative controls confirmed that there was no detectable contamination. Therefore, only the samples from the neonicotinoid treatment and the combined treatment were used in the subsequent analysis. Consequently, each substance (concentration per gram dry mass) entered the model as a response variable together with caste and virus treatment as fixed effects and colony identity as a random factor to respect that both the queen and workers of each colony were tested. For pairwise comparisons, we then used Wilcoxon signed-rank tests respecting when queens and workers of the same colonies were compared (paired = TRUE). The ratio of clothianidin to thiamethoxam was calculated for all samples positive for both substances (queens N = 15, workers N = 16). Then, the ratio was log-transformed and analysed using a linear mixed-effect model with caste as a fixed effect and treatment as well as colony identity as random factors.

The variables of the behavioural assay overall movement and average speed were analysed with linear mixed effect models, while time inactive and initial speed each entered a Poisson linear mixed effect model due to the nature of their data, which reflects counts with large values being rare events, i.e. Poisson distribution^91^. In all cases, both treatments and their interaction were used as fixed effects with recording date and colony identity as random factors to control for potential effects of the recording day and measurements of five workers per colony that should not be regarded as independent measurements.

### Data transparency appendix

Parts of the data reported in this manuscript have been previously published in two other manuscripts. The first publication focused on foodborne transmission and clinical symptoms of honey bee viruses in ants and contained, among other data, the two neonicotinoid control groups reported in this manuscript^48^. In the second publication, the long-term impact of chronic neonicotinoid exposure on ant colony development was investigated^22^, based on the two neonicotinoid groups reported in this manuscript. Here, although we confirm the findings of the previous studies with the expanded dataset, we focus on the interaction of the two factors thiamethoxam and ABPV, to provide an independent contribution to the ongoing debate about stressor interactions. Due to the special focus on stressor interactions, the overlap is mostly restricted to the material and methods section.

## Supplementary Information

Supplementary Information.

## Data Availability

The complete raw data and a statistics-summary table is available on figshare and it can be accessed using the following digital object identifier (DOI): 10.6084/m9.figshare.14071373.
