# Supplementary material for: Varying impact of neonicotinoid insecticide and acute bee paralysis virus across castes and colonies of black garden ants, Lasius niger (Hymenoptera: Formicidae)
Source: Sci Rep. 2021 Oct 15;11:20500. doi: 10.1038/s41598-021-98406-w (PMC8519937; doi:10.1038/s41598-021-98406-w)
Supplement: Supplementary file 1 — Supplementary Information. [file 41598_2021_98406_MOESM1_ESM.docx]

**Supplementary material**

Varying impact of neonicotinoid insecticide and acute bee paralysis virus across castes and colonies of black garden ants, *Lasius niger* (Hymenoptera: Formicidae)

Daniel Schläppi^1,2^*, Nina Kettler^1^, Gaétan Glauser^3^, Lars Straub^1,4^, Orlando Yañez^1,4^, Peter Neumann^1,4^

^1^ Institute of Bee Health, Vetsuisse Faculty, University of Bern, Bern, Switzerland

^2^ School of Biological Sciences, University of Bristol, Bristol, United Kingdom

^3^ Neuchâtel Platform of Analytical Chemistry, University of Neuchâtel, Neuchâtel, Switzerland

^4^ Agroscope, Swiss Bee Research Centre, Bern, Switzerland

*Corresponding author: d.schlaeppi@mail.ch

# Abstract

Pesticides and pathogens are known drivers of declines in global entomofauna. However, interactions between pesticides and viruses, which could range from antagonistic, over additive to synergistic, are poorly understood in ants. Here, we show that in ants the impact of single and combined pesticide and virus stressors can vary across castes and at the colony level. A fully-crossed laboratory assay was used to evaluate interactions between a sublethal dose of the neonicotinoid thiamethoxam and Acute bee paralysis virus (ABPV) in black garden ants, Lasius niger. After monitoring colonies over 64 weeks, body mass, neonicotinoid residues and virus titres of workers and queens, as well as worker behavioural activity were measured. ABPV, but not thiamethoxam, reduced activity of workers. Neonicotinoid exposure resulted in reduced body mass of workers, but not of queens. Further, thiamethoxam can facilitate ABPV infections in queens, but not in workers. Overall, virus exposure did not compromise detoxification and body mass, but one colony showed high virus titres and worker mortality. Although the data suggest additive effects at the level of individuals and castes, co-exposure with both stressors elicited antagonistic effects on colony size. Our results create demand for long-term holistic risk assessment of individual stressors and their interactions to protect biodiversity.

Keywords: ant, clothianidin, neonicotinoid, pathogen, stressor interaction, thiamethoxam, virus

**Supplementary Figure 1:** Experimental timeline - Chronological order of events starting with the collection of queens (Week 0) until termination (week 64), including the death of queens (†) which were assigned to one of four treatment groups: (i) Controls, (ii) neonicotinoid = chronic exposure to thiamethoxam (30 μg/L), (iii) virus = feeding regime with Acute bee paralysis virus (ABPV), (iv) combined = exposure to thiamethoxam and ABPV.

**Supplementary Table 1:** Chronological order of the main events of the experiment with dates and the number of days or weeks that passed since the collection of the queens for each event, including the death of queens (†), which were assigned to one of four treatment groups: (i) Controls, (ii) neonicotinoid = chronic exposure to thiamethoxam (30 μg/L), (iii) virus = feeding regime with Acute bee paralysis virus (ABPV), (iv) combined = exposure to thiamethoxam and ABPV.

| **Date** | **Day** | **Week** | **Event** |
| --- | --- | --- | --- |
| 30.07.16 | 0 | 0 | Collection of gynes in the field |
| 11.08.16 | 12 | 2 | Initiation of the experiment with the transfer into the nesting tubes |
| 14.08.16 | 15 | 3 | †Queen (control) |
| 26.08.16 | 27 | 4 | †Queen (neonicotinoid) |
| 23.09.16 | 55 | 8 | †Queen (combined) |
| 09.11.16 | 102 | 15 | Start of the overwintering in a fridge at 6 °C (2-week acclimatisation period 14 °C before and after overwintering)  32 weeks with a 2-week acclimatisation period at 14 °C before and after  overwintering. |
| 03.03.17 | 216 | 31 | End of the overwintering |
| 28.03.17 | 241 | 34 | Translocation into new nesting tubes with an attached foraging arena |
| 10.05.17 | 284 | 41 | †Queen (combined) |
| 17.05.17 | 291 | 42 | †Queen (control) |
| 25.05.17 | 299 | 47 | †Queen (virus) |
| 11.07.17 | 346 | 50 | †Queen (neonicotinoid) |
| 16.07.17 | 351 | 51 | Start of the feeding regime with honey bee pupae (*Apis mellifera*) |
| 30.08.17 | 396 | 57 | †Queen (virus) |
| 26.09.17 | 423 | 61 | End of the feeding regime |
| 16.10.17 | 443 | 64 | End of the Experiment by freezing all colonies at -80°C |

**
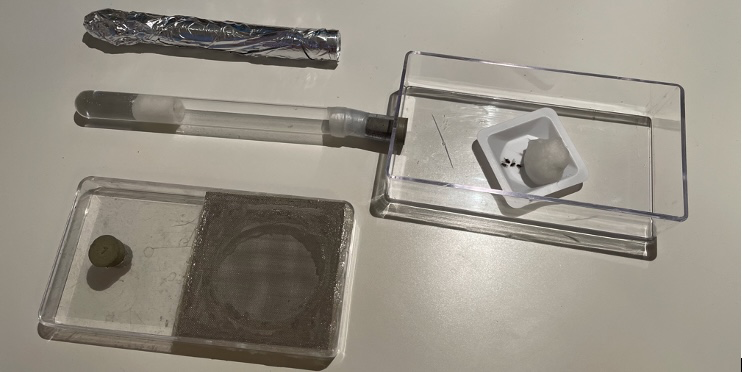
**

**Supplementary Figure 2:** Experimental cages consisting of a nesting tube (length = 155 mm, diameter = 14 mm) attached to a foraging arena (135x68x32 mm). The nesting tube was separated into two compartments with a cotton wool ball. The hind chamber was filled with water (controls) or thiamethoxam treatment solution (30 μg/L). The lid of the foraging arena can be removed to access the inside for feeding (weekly provision of a sugar-water (40% mass fraction of sugar) drenched cotton ball and four fruit flies (*Drosophila hidey*) or honey bee pupae (*Apis mellifera*) on a small bowl).
